# Co-existence of *bla*_OXA-23_ and *bla*_NDM-1_ genes of *Acinetobacter baumannii* isolated from Nepal: antimicrobial resistance and clinical significance

**DOI:** 10.1186/s13756-017-0180-5

**Published:** 2017-02-07

**Authors:** Prabhu Raj Joshi, Mahesh Acharya, Trishna Kakshapati, Udomluk Leungtongkam, Rapee Thummeepak, Sutthirat Sitthisak

**Affiliations:** 1 Central Department of Microbiology, Tribhuvan University, Kirtipur, Kathmandu Nepal; 2 Annapurna Neurological Institute and Allied Sciences, Maitighar, Kathmandu Nepal; 3 Department of Microbiology and Parasitology, Faculty of Medical Science, Naresuan University, Phitsanulok, Thailand; 4 Centre of Excellence in Medical Biotechnology, Faculty of Medical Science, Naresuan University, Phitsanulok, Thailand

**Keywords:** *Acinetobacter baumannii*, Carbapenem resistance, *bla*_OXA-23_ and *bla*_NDM-1_ carbapenemase genes

## Abstract

**Background:**

Molecular analysis of carbapenem-resistant genes in *Acinetobacter baumannii*, an emerging pathogen, is less commonly reported from Nepal. In this study we determined the antibiotic susceptibility profile and genetic mechanism of carbapenem resistance in clinical isolates of *A. baumannii.*

**Methods:**

*A. baumannii* were isolated from various clinical specimens and identified based on Gram staining, biochemical tests, and PCR amplification of organism specific 16S rRNA and *bla*_OXA-51_ genes. The antibiotic susceptibility testing was performed using disc diffusion and E-test method. Multiplex PCR assays were used to detect the following β-lactamase genes: four class D carbapenem hydrolyzing oxacillinases (*bla*_OXA-51_, *bla*_OXA-23_, *bla*_OXA-24_ and *bla*_OXA-58_). Uniplex PCRs were used to detect three class B metallo-β-lactamases genes (*bla*_IMP_, *bla*_VIM_ and *bla*_NDM-1_), class C cephalosporin resistance genes (*bla*_ADC_), aminoglycoside resistance gene (*aphA6*), and IS*Aba1* of all isolates. Insertion sequence IS*Aba125* among NDM-1 positive strains was detected. Clonal relatedness of all isolates were analyzed using repetitive sequence-based PCR (rep-PCR).

**Results:**

Of total 44 analyzed isolates, 97.7% (*n* = 43) were carbapenem-resistant *A. baumannii* (CR-AB) and 97.7% (*n* = 43) were multidrug resistant *A. baumannii* (MDR-AB). One isolate was detected to be extremely drug resistant *A. baumannii* (XDR-AB). All the isolates were fully susceptible to colistin (MICs < 2 μg/ml). The *bla*_OXA-23_ gene was detected in all isolates, while *bla*_NDM-1_ was detected in 6 isolates (13.6%). Insertion sequence, IS*Aba1* was detected in all of *bla*_OXA-23_ positive isolates. IS*Aba125* was detected in all *bla*_NDM-1_ positive strains. The *bla*_ADC_ and *aphA6* genes were detected in 90.1 and 40.1%, respectively. The rep-PCR of all isolates represented 7 different genotypes.

**Conclusion:**

We found high prevalence of CR-AB and MDR-AB with *bla*_OXA-23_ gene in a tertiary care hospital in Nepal. Systemic network surveillance should be established for monitoring and controlling the spread of these resistant strains.

**Electronic supplementary material:**

The online version of this article (doi:10.1186/s13756-017-0180-5) contains supplementary material, which is available to authorized users.

## Background

*Acinetobacter baumannii*, an emerging pathogen of healthcare centers, shows intrinsic as well as acquired drug-resistance mechanisms [1]. Multidrug-resistant *A. baumannii* can be resistant to all of the currently available antibiotics, and in its deadliest form these are only susceptible to potentially toxic polymyxins and colistins, leaving limited options for treatment [2]. Infections with carbapenem- and colistin-resistant *A. baumannii* are emerging globally [3].

Carbapenem resistance in *A. baumannii* encompasses production of class B, C and class D carbapenemase, decreased membrane permeability, altered penicillin-binding proteins, and overexpression of efflux pumps [4, 5]. Most commonly, *Acinetobacter* spp. develop carbapenem resistance by production of OXA-type carbapenemase and metallo-β-lactamases (MBLs) [6, 7]; *bla*_OXA-23_-like, *bla*_OXA-40_-like, *bla*_OXA-58_-like and *bla*_OXA-51_-like carbapenemases are broadly reported, where *bla*_OXA-51_-like β-lactamases, intrinsic to *A. baumannii*, is used for species identification [8–10]. Among multiple MBL genes, *bla*_IMP_ and *bla*_VIM_ types (chromosomal or plasmid encoded) encode carbapenemase in *A. baumannii* [9]. *A. baumannii* harboring plasmid encoded New Delhi metallo-β-lactamase-1 (NDM-1), a novel carbapenemase gene, is reported from many countries [11, 12]. In addition, detection of class C β-lactamase genes (*bla*_ADC_) which mediated cephalosporin resistances and aminoglycoside resistant genes (*aphA6*) has increased in recent years in *A. baumannii* clinical isolates [13, 14].

*A. baumannii* remains a critical problem in many healthcare settings throughout the world despite the implementation of infection control practices. There are limited data on carbapenem-resistant *A. baumannii* in Nepal. The objective of this study was to determine antibiotic susceptibility profile, antibiotic resistance genes and genetic mechanism of carbapenem resistance of *A. baumannii* in clinical isolates at a tertiary care hospital, Nepal.

## Methods

### Bacterial isolation and identification

*A. baumannii* isolates were collected from inpatient units of a tertiary hospital, Nepal. Forty-four non-duplicate isolates were collected (24 male and 20 female; age range between 24 to 80 years) over 9 months periods (October 2014 to June 2015). All isolates were identified by classical biochemical methods and confirmed by PCR method for detecting 16S rRNA gene and *bla*_OXA-51_ gene [15, 16]. Isolates were identified as *A. baumannii* by PCR result of positive for both PCRs.

### Antibiotic susceptibility testing

The antibiotic susceptibility of amikacin (30), cefotaxime (30), ceftazidime (30), ceftriaxone (30), cefepime (30), ciprofloxacin (5), gentamicin (10), imipenem (10), meropenem (10), trimethoprim/sulfamethoxazole (1.25/23.75), tetracycline (30), and piperacillin/tazobactam (100/10) (Oxoid) was determined on Mueller Hinton Agar (High Media, India) according to the antibiotic disk diffusion method [17]. The plates were incubated at 37 °C for 24 h. The zones of inhibition were determined whether the microorganism was susceptible, intermediately resistant, or resistant to each antibiotic according to Clinical and Laboratory Standards Institute (CLSI) guidelines. E-test was performed to determine the Minimum inhibitory concentration (MIC) of ceftazidime, imipenem, tigecycline and colistin (High Media, India) according to manufacturer instructions and interpreted as per CLSI guidelines except for tigecycline. Multidrung-resistant *A. baumannii* (MDR-AB) was defined when *A. baumannii* resistant to multiple antibiotics, often defined as three or more antibiotic classes. Extensively drug resistant *A. baumannii* (XDR-AB) was defined when *A. baumannii* was resistant to all antimicrobial agents except polymyxins (colistin) [18].

### PCR amplification of antibiotic resistance genes

PCR assays to detect *bla*_OXA-23_, *bla*_OXA-24_, *bla*_OXA-51_, *bla*_OXA-58,_ *bla*_IMP,_ *bla*_VIM*,*_ *bla*_NDM,_ *bla*_ADC_ and *ahpA6* genes were performed using primers as describe previously (Table 1). The amplification reaction was performed using *A. baumannii* cell lysate as DNA template. Each PCR was performed in triplicate in a thermocycler with a PCR condition as described previously [14, 16, 19–21]. All PCR assays used 16S rRNA or *bla*_OXA-51_ genes as the internal control. The IS*Aba1* of *bla*_OXA-23_ gene was detected using combination of primers IS*Aba1*-F/IS*Aba1*-R and IS*Aba1*-F*/bla*_OXA-23_-R (Table 1) [22]. The IS*Aba125* of *bla*_NDM-1_ gene were determined in all *bla*_NDM-1_ positive strains using combination of primers ISA125-F/ISA125-R and ISA125-F/*bla*_NDM_-R (Table 1). PCR products of the *bla*_NDM-1_ genes were purified and sequenced. BLAST was used to compare the sequences of *bla*_NDM-1_ genes against the GenBank Database. PCR products were analyzed by electrophoresis in 1% agarose gel containing 0.5 μg/ml ethidium bromides.

**Table 1:** List of primer for detection of genes used in this study

Target genes	Primer name	Sequence 5’-3’	Size/ Annealing temp.	References
16S rRNA	16S rRNA-F	AGAGTTTGATCCTGGCTCAG	1500/58	[15]
	16S rRNA-R	ACGGCTACCTTGTTACGACTT		
*bla*_OXA-23_	*bla*_OXA-23_-F	GATCGGATTGGAGAACCAGA	501/52	[16]
	*bla*_OXA-23_-R	ATTTCTGACCGCATTTCCAT		
*bla*_OXA-51_	*bla*_OXA-51_-F	TAATGCTTTGATCGGCCTTG	353/52	[16]
	*bla*_OXA-51_-R	TGGATTGCACTTCATCTTGG		
*bla*_OXA-24_	*bla*_OXA-24_-F	GGTTAGTTGGCCCCCTTAAA	246/52	[16]
	*bla*_OXA-24_-R	AGTTGAGCGAAAAGGGGATT		
*bla*_OXA-58_	*bla*_OXA-58_-F	AAGTATTGGGGCTTGTGCTG	599/52	[16]
	*bla*_OXA-58_-R	CCCCTCTGCGCTCTACATAC		
*bla*_IMP_	*bla*_IMP_ –F	GGAATAGAGTGGCTTAAYTCTC	232/52	[20]
	*bla*_IMP_ –R	GGTTTAAYAAAACAACCACC		
*bla*_VIM_	*bla*_VIM_ –F	GATGGTGTTTGGTCGCATA	390/52	[20]
	*bla*_VIM_ –R	CGAATGCGCAGCACCAG		
*bla*_NDM_	*bla*_NDM_–F	GGTTTGGCGATCTGGTTTTC	621/52	[21]
	*bla*_NDM_–R	CGGAATGGCTCATCACGATC		
*bla*_ADC_	*bla*_ADC_-F	TAAACACCACATATGTTCCG	663/56	[19]
	*bla*_ADC_-F	ACTTACTTCAACTCGCGACG		
*aphA6*	*aphA6*-F	ATGGAATTGCCCAATATTATTC	736/55	[14]
	*aphA6*-R	TCAATTCAATTCATCAAGTTTTA		
IS*Aba1*	IS*Aba1*-F	CATTGGCATTAAACTGAGGAGAAA	451/52	[22]
	IS*Aba1*-R	TTGGAAATGGGGAAAACGAA		
IS*Aba125*	ISA125-F	TGTTGAAGCGATCCGTTGTT	755/57	This study
	ISA125-R	GTGCGACAGTTTCAAAAGCCA		
Rep-PCR	ERIC-2	AAGTAAGTGACTGGGGTGAGCG	variable length/45	[24]

### IPM-EDTA combined disk test

All *bla*_NDM-1_ positive strains were tested for MBL production by IPM-EDTA combined disk test. The test was performed as previously described [23]. After 24 h incubation, the difference of inhibition zone diameter between IPM-EDTA disk and IPM disk alone (≥7 mm) was considered the positive criteria for the presence of MBL.

### Repetitive element PCR-mediated DNA fingerprinting (rep-PCR)

Genomic DNA of each isolates was extracted from the overnight cultures using GF-1 bacterial DNA extraction kit (Vivantis, Malaysia). Rep-PCR was performed by using genomic DNA as a template for PCR amplification with the ERIC-2 primer (Table 1) using condition as describe previously [24, 25]. PCR-banding patterns and rep-PCR types were analyzed and interpreted as previously described [25].

## Results

### Demographic characteristic of patients

**Fig. 1:**
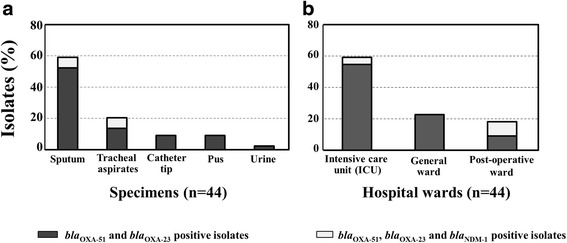
Distribution of *A. baumannii* carrying carbapenemase genes in different specimen types **a** and wards **b**

Demographic characteristics of the inpatients with *A. baumannii* infection were analyzed; 24 (54.5%) were male and 20 (45.5%) were female. Most of the specimens were from ICU wards (*n* = 27, 61.4%) (Fig. 1). Isolates were collected from sputum (*n* = 26, 59.1%), tracheal aspirates (*n* = 9, 20.4%), catheter tip, (*n* = 4, 9.1%), pus (*n* = 4, 9.1%) and urine (*n* = 1, 2.3%) (Fig. 1).

### Antibiotic susceptibility

Of the 44 isolates, resistance was found against ciprofloxacin (*n* = 43, 97.7%), cefotaxime (*n* = 43, 97.7%), ceftazidime (*n* = 42, 95.4%), ceftriaxone (*n* = 41, 93.2%), cefepime (*n* = 39, 88.6%), amikacin (*n* = 19, 43.2%), gentamicin (*n* = 23, 52.3%), trimethoprim/sulfamethoxazole (*n* = 41, 93.2%), tetracycline (*n* = 21, 47.7%) and piperacillin/tazobactam (*n* = 43, 97.7%). Only one isolate of *A.baumannii* was susceptible to all tested antibiotics. Most isolates (97.7%, *n* = 43) were carbapenem resistant *A. baumannii* (CR-AB); all CR-AB were MDR-AB. One isolate was detected to be XDR-AB. All the isolates were fully susceptible to colistin (MICs < 2 μg/ml) and MIC of tigecycline was determined to be <2.5 μg/ml (Table 2).

**Table 2:** The carbapenemases gene patterns, rep-PCR types and MIC determination of *A. baumannii* isolated from difference wards

Sites	β-lactamase gene patterns	No. of isolates	Rep-PCR Types (*n*)	MIC (μg/ml) range			
				CAZ	IMP	TG	CL
Intensive care unit	*bla*_OXA-51/ OXA-23_	25	A (2), B (6), C (8), D (6), E (2), F (1)	4– > 256	1– > 32	1.6–3.9	0.13–2
	*bla*_OXA-51/ OXA-23/ NDM-1_	2	B (1), C (1)	>256	>32	1.7–3.2	0.61–0.79
General ward	*bla*_OXA-51/ OXA-23_	9	A (1), B (1), C (1), D (5), G (1)	>256	>24–>32	2–3.4	0.32–0.88
Post-operative ward	*bla*_OXA-51/ OXA-23_	4	A (1), B (1), C (2)	>256	>32	2.3–3.3	0.54–0.78
	*bla*_OXA-51/ OXA-23/ NDM-1_	4	A (1), C (2) D (1)	>256	>32	2.1–3.2	0.23–0.51

*Abbreviations*: *CAZ* ceftazidime, *IPM* imipenem, *TG* tigecycline, *CL* colistin

### Antibiotic resistance genes and IS element in *A. baumannii*

Aminoglycoside resistance gene, *aphA6* and cephalosporin resistance genes, *bla*_ADC_ were detected in 40.1% (18/44) and 90.1% (40/44), respectively. The *bla*_OXA-23_ was present in all isolates. Other class D β-lactamase genes, including *bla*_OXA-24_ and *bla*_OXA-58_, markers of carbapenem resistance in *A. baumannii*, were not detected in analyzed isolates. IS*Aba1* was detected in all of *bla*_OXA-23_ positive isolates (100%). Of total analyzed isolates, 6 (13.6%) also harbored *bla*_NDM-1_ gene in addition to *bla*_OXA-23_ and *bla*_OXA-51_. All NDM-1 positive strains exhibited insertion sequence IS*Aba125* detecting with primers ISA125-F/ISA125-R. All isolates also detected a band of 1.6 kb in a PCR using ISA125-F/*bla*_NDM_-R primers. Metallo-β-lactamase (MBL) genes, including *bla*_VIM_ and *bla*_IMP_, were not detected in all isolates. The sequences of the *bla*_NDM-1_ gene yielded 99-100% sequence identity to the *bla*_NDM-1_ gene from *Acinetobacter lwoffii* strain WJ10621 plasmid pNDM-BJ01 (Accession: JQ001791) obtained from the GenBank Database.

### MBL production

Six *A. baumannii* isolates harbored *bla*_NDM-1_ gene were detected for MBL production. All of *bla*_NDM-1_ positive strains were positive for MBL production. MBL positive strains showed resistance to fluoroquinolones and β-lactam.

### Epidemiological typing

**Fig. 2:**
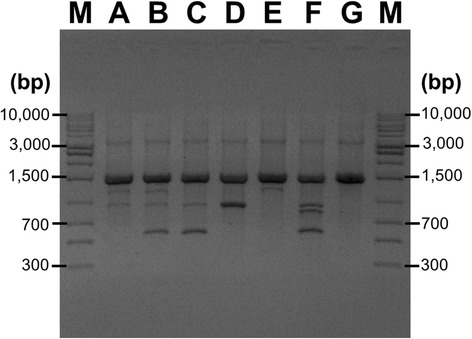
Rep-PCR-based DNA fingerprint patterns of *A. baumannii* isolates. The *lanes* marked M contain molecular markers. Each lane represents genotype patterns of *A*–*G*

Clonal relationship among isolates were studied using rep-PCR typing. The fingerprinting represented 7 different DNA patterns consisting of 2 to 5 DNA fragment sizes. The amplicons size for ERIC-2 PCR was 500–4000 bp. The genotype was named A-G as shown in Fig. 2. The high prevalence genotype was type C (*n* = 14; 31.8%) and D (*n* = 12; 27.3%). Genotype A, B, C and D were disseminated in all isolated ward (ICU, general ward and post-operative ward). Among 44 isolates, one isolate of type F (2.3%) and G (2.3%) was found. Type F was obtained from a catheter tip specimen from the ICU ward. Type G was obtained from sputum of a patient from a general ward. All NDM-1 positive strains exhibited genotype A (*n* = 1), B (*n* = 1), C (*n* = 3) and D (*n* = 1).

## Discussions

*A. baumannii* harboring *bla*_OXA-51_-like gene has been identified as a marker for species identification. An intrinsic *bla*_OXA-51_-like gene detected in all isolates in this study supports the use of this gene as a surrogate marker of *A. baumannii* identification [8–10]. High prevalence of cephalosporin resistance genes, *bla*_ADC_ (90.1%) was found in this study. In addition, we found a high rate of cepharosporin resistant antibiotics (cefotaxime, ceftazidime, ceftriaxone) using the disk diffusion method. These data indicated that cephalosporins no longer work to treat *A. baumannii* isolated from Nepal. Carbapenem resistance in *A. baumannii* is a major concern and is most often associated with class D β-lactamases and MBLs. The full susceptibility of all CR-AB to colistin in this study indicates that colistin is still an option of drug for the treatment of infections caused by *A. baumannii* in Nepal hospital.

OXA-type carbapenemases are predominant in *A. baumannii* [6, 7]. In agreement with this finding, high prevalence of *bla*_OXA-23_ carrying *A. baumannii* strains has been reported in Nepalese patients [26]. The acquired *bla*_OXA-23_ is the dominant genetic determinant in Asia. The *bla*_OXA-23_ gene located on plasmid can be transferred between *A. baumannii* through conjugation. Thus, antibiotic resistant bacteria have been rapidly increasing worldwide [27]. The *bla*_OXA-24_ and *bla*_OXA-58_ were not detected in any isolates from this study. The *bla*_OXA-24/40_ and *bla*_OXA-58_ genes were common in *A. baumannii* isolated from Europe [2, 28]. Recently, *bla*_OXA-143_ and *bla*_OXA-235_, which are novel class D β-lactamase genes in *A. baumannii* have been identified. To date, these determinants were detected only in Brazil, Mexico and the USA [29, 30]. IS*Aba1* was detected in widespread clones of *A. baumannii* worldwide. Our study found IS*Aba1* upstream of *bla*_OXA-23_ in all *A. baumannii* isolates. A correlation between *A. baumannii* clusters carrying the IS*Aba1*/*bla*_OXA-23_ gene and increased minimal inhibitory concentrations for carbapenems was reported [31]. One isolate (AB-13) that was recovered from catheter tips of long-stay hospital patients showed an extreme drug resistance pattern (Additional file 1: Table S1). This isolate represented *bla*_OXA-23_, *bla*_ADC_ and *aphA6* genes. Further molecular study to detect other antibiotic resistance genes is needed to explain what factors correlated with extreme drug resistance. We also found one isolate (AB-25) harboring *bla*_OXA-23_, *bla*_ADC_ and *aphA6* genes was sensitive to all tested drugs (Additional file 1: Table S1). This may be due to the lack of promoter or mutation of IS*Aba1* or *bla*_OXA-23_ gene_._ Further study is needed to warrant the conclusion.

The *bla*_NDM-1_ carrying *A. baumannii* has recently been emerged in many countries, including Germany, Spain, Israel, Egypt, Switzerland, Libya, India, Pakistan and Nepal [11, 26, 32, 33]. The *bla*_NDM-1_ gene has been identified as a chimeric gene constructed by the fusion of the aminoglycoside-resistance gene *aphA6* with a mannose-binding lectin gene. This event most likely occurs in *Acinetobacter* spp., indicating that these bacteria are likely the origin of this gene [34]. In this study, we identified 13.6% of *A. baumannii* carrying *bla*_NDM-1_ gene. Previous study has identified high prevalence (24.6%) of the *A. baumannii* harbored the *bla*_NDM-1_ gene in Nepal in 2013–2014 [26]. Taking into consideration the relationship between India, China and Nepal, the spread of *bla*_NDM-1_ is likely to occur rapidly, mostly through *A. baumannii* rather than Enterobacteriaceae. *A. baumannii* able to transfer the *bla*_NDM-1_ gene via conjugation to the recipients and Tn*125* appears to be the main vehicle for dissemination of the *bla*_NDM-1_ genes in *A. baumannii* [35]. Poirel et al. reported that the *bla*_NDM-1_ gene was located within the composite transposon Tn*125* bracketed by two copies of a strong promoter of *bla*_NDM-1_ gene called IS*Aba125* [11]. This report was correlated with our finding that found IS*Aba125* in 100% of NDM-1 producing *A. baumannii*.

The previous study reported that the most of *A. baumannii* isolates harboring *bla*_NDM-1_ belonged to ST85 and ST25 [35–37]. In Libyan hospital, Libya, the main clone of imipenem-resistant NDM-1-producing *A. baumannii* belonged to ST2 [33]. We used rep-PCR typing to determine the clonal relationship in NDM-1 producing *A. baumannii*. Our study highlighted that most of NDM-1-producing *A. baumannii* isolates belonged to 4 genotypes using rep-PCR. Rep-PCR is a method that generates DNA fingerprints to discriminate between bacterial strains, and has been used to characterize *A. baumannii* isolates from hospitalized patients [38]. Our rep-PCR typing represented a high genetic diversity (A-G) among *A. baumannii* isolates from Nepal. Some clonally related groups (A, B, C and D) were observed in the all wards represented the disseminated of these clones in the hospital. Four genotypes (A, B, C, and D) of co-existence of *bla*_OXA-23_ and *bla*_NDM-1_ *A. baumannii* isolates were found. In addition, dissemination of these four genotypes into different wards also confirms as a major epidemic. Since rep-PCR is less discriminatory for molecular typing of bacterial strains, further study using multi-locus sequence typing could be useful for epidemiological investigations.

## Conclusion

Antibiotic resistance in *A. baumannii* is considered to be a major future challenge in Nepal. Beyond OXA-type carbapenemase, there is no doubt the emergence and spreads of NDM-1 encoding *A. baumannii*–a superbug–will further limit chemotherapeutic options and threaten the public health of Nepal. The mechanism of hospital adaptiveness beyond antibiotic resistance will be more demanded in order to fully understand and combat MDR and XDR *A. baumannii*.

## Additional file

Additional file 1: Table S1. Type of clinical specimen, ward, antibiotic susceptibility patterns, rep-PCR types, resistance genes and MIC of 44 *A. baumannii* isolates. (DOCX 23 kb)

## Abbreviations

CR-AB: Carbapenem-resistant *Acinetobacter baumannii*
ICU: Intensive care unit
IPM-EDTA: Imipenem-ethylenediaminetetraacetic acid
MBL: Metallo-beta-lactamase
MDR-AB: Multidrug-resistant *Acinetobacter baumannii*
MIC: Minimum inhibitory concentration
NDM: New Delhi metallo-beta-lactamase
PCR: Polymerase chain reaction
XDR-AB: Extremely drug resistant *Acinetobacter baumannii*

## References

[1] Yamamoto M, Nagao M, Matsumura Y, Matsushima A, Ito Y, Takakura S (2011). Interspecies dissemination of a novel class 1 integron carrying *bla*_IMP-19_ among *Acinetobacter* species in Japan. J Antimicrob Chemother.

[2] Villalon P, Valdezate S, Medina-Pascual MJ, Carrasco G, Vindel A, Saez-Nieto JA (2013). Epidemiology of the *Acinetobacter*-derived cephalosporinase, carbapenem-hydrolysingoxacillinase and metallo-beta-lactamase genes, and of common insertion sequences, in epidemic clones of *Acinetobacter baumannii* from Spain. J Antimicrob Chemother.

[3] Agodi A, Voulgari E, Barchitta M, Quattrocchi A, Bellocchi P, Poulou A (2014). Spread of a carbapenem- and colistin-resistant *Acinetobacter baumannii* ST2 clonal strain causing outbreaks in two Sicilian hospitals. J Hosp Infect.

[4] Heritier C, Poirel L, Lambert T, Nordmann P (2005). Contribution of acquired carbapenem-hydrolyzing oxacillinases to carbapenem resistance in *Acinetobacter baumannii*. Antimicrob Agents Chemother.

[5] Quale J, Bratu S, Landman D, Heddurshetti R (2003). Molecular epidemiology and mechanisms of carbapenem resistance in *Acinetobacter baumannii* endemic in New York City. Clin Infect Dis Off Publ Infect Dis Soc Am.

[6] Amudhan MS, Sekar U, Kamalanathan A, Balaraman S (2012). *bla*_IMP_ and *bla*_VIM_ mediated carbapenem resistance in *Pseudomonas* and *Acinetobacter* species in India. J Infect Develop Ctries.

[7] Thomson JM, Bonomo RA (2005). The threat of antibiotic resistance in Gram negative pathogenic bacteria: beta-lactams in peril!. Curr Opin Microbiol.

[8] Cicek AC, Saral A, Iraz M, Ceylan A, Duzgun AO, Peleg AY (2014). OXA- and GES-type beta-lactamases predominate in extensively drug-resistant *Acinetobacter baumannii* isolates from a Turkish University Hospital. Clin Microbiol Infect.

[9] Tsakris A, Ikonomidis A, Pournaras S, Tzouvelekis LS, Sofianou D, Legakis NJ (2006). VIM-1 metallo-beta-lactamase in *Acinetobacter baumannii*. Emerg Infect Dis.

[10] Kusradze I, Diene SM, Goderdzishvili M, Rolain JM (2011). Molecular detection of OXA carbapenemase genes in multidrug-resistant *Acinetobacter baumannii* isolates from Iraq and Georgia. Int J Antimicrob Agents.

[11] Poirel L, Bonnin RA, Boulanger A, Schrenzel J, Kaase M, Nordmann P (2012). Tn125-related acquisition of *bla*NDM-like genes in *Acinetobacter baumannii*. Antimicrob Agents Chemother.

[12] Abbas M, Cherkaoui A, Fankhauser C, Schrenzel J, Harbarth S (2012). Epidemiology and clinical implications of carbapenemase-producing bacteria in Switzerland. Rev Med Suisse.

[13] Sarhaddi N, Soleimanpour S, Farsiani H, Mosavat A, Dolatabadi S, Salimizand H (2016). Elevated prevalence of multidrug-resistant *Acinetobacter baumannii* with extensive genetic diversity in the largest burn centre of northeast Iran. J Glob Antimicrob Resist.

[14] Hujer KM, Hujer AM, Hulten EA, Bajaksouzian S, Adams JM, Donskey CJ (2006). Analysis of antibiotic resistance genes in multidrug-resistant *Acinetobacter* sp. isolates from military and civilian patients treated at the Walter Reed Army Medical Center. Antimicrob Agents Chemother.

[15] Misbah S, Hassan H, Yusof MY, Hanifah YS, Abu-Bakar S (2005). Genomic species identification of *Acinetobacter* of clinical isolates by 16S rDNA sequencing. Singapore. Med J.

[16] Woodford N, Ellington MJ, Coelho JM, Turton JF, Ward ME, Brown S (2006). Multiplex PCR for genes encoding prevalent OXA carbapenemases in *Acinetobacter* spp. Int J Antimicrob Agents.

[17] Clinical and Laboratory Standards Institute (2014). Performance standards for antimicrobial susceptibility testing, document M100-24.

[18] Magiorakos AP, Srinivasan A, Carey RB, Carmeli Y, Falagas ME, Giske CG (2012). Multidrug-resistant, extensively drug-resistant and pandrug-resistant bacteria: an international expert proposal for interim standard definitions for acquired resistance. Clin Microbiol Infect.

[19] Liu Y, Liu X (2015). Detection of AmpC β-lactamases in *Acinetobacter baumannii* in the Xuzhou region and analysis of drug resistance. Exp Ther Med.

[20] Ellington MJ, Kistler J, Livermore DM, Woodford N (2007). Multiplex PCR for rapid detection of genes encoding acquired metallo-beta-lactamases. J Antimicrob Chemother.

[21] Poirel L, Walsh TR, Cuvillier V, Nordmann P (2011). Multiplex PCR for detection of acquired carbapenemase genes. Diagn Microbiol Infect Dis.

[22] Ruiz M, Marti S, Fernandez-Cuenca F, Pascual A, Vila J (2007). Prevalence of IS (*Aba1*) in epidemiologically unrelated *Acinetobacter baumannii* clinical isolates. FEMS Microbiol Lett.

[23] Yong D, Lee K, Yum JH, Shin HB, Rossolini GM, Chong Y (2002). Imipenem-EDTA disk method for differentiation of metallo-beta-lactamase-producing clinical isolates of *Pseudomonas* spp. and *Acinetobacter* spp. J Clin Microbiol.

[24] Vila J, Marcos MA, Jiminez de Anta MT (1996). A comparative study of different PCR-based DNA fingerprinting techniques for typing of the *Acinetobacter calcoaceticus-A. baumannii* complex. J Med Microbiol.

[25] Grundmann HJ, Towner KJ, Dijkshoorn L, Gerner-Smidt P, Maher M, Seifert H (1997). Multicenter study using standardized protocols and reagents for evaluation of reproducibility of PCR-based fingerprinting of *Acinetobacter* spp. J Clin Microbiol.

[26] Shrestha S, Tadab T, Miyoshi-Akiyamac T, Oharad H, Shimadab K, Satoue K (2015). Molecular epidemiology of multidrug-resistant *Acinetobacter baumannii* isolates in a university hospital in Nepal reveals the emergence of a novel epidemic clonal lineage. Int J Antimicrob Agents.

[27] Bertini A, Poirel L, Mugnier PD, Villa L, Nordmann P, Carattoli A (2010). Characterization and PCR-based replicon typing of resistance plasmids in *Acinetobacter baumannii*. Antimicrob Agents Chemother.

[28] Cherkaoui A, Emonet S, Renzi G, Schrenzel J (2015). Characteristics of multidrug-resistant *Acinetobacter baumannii* strains isolated in Geneva during colonization or infection. Ann Clin Microbiol Antimicrob.

[29] Higgins PG, Pérez-Llarena FJ, Zander E, Fernández A, Bou G, Seifert H (2013). OXA-235, a novel class D β-lactamase involved in resistance to carbapenems in *Acinetobacter baumannii*. Antimicrob Agents Chemother.

[30] Zander E, Bonnin RA, Seifert H, Higgins PG (2014). Characterization of *bla*_OXA-143_ variants in *Acinetobacter baumannii* and *Acinetobacter pittii*. Antimicrob Agents Chemother.

[31] Viana GF, Zago MC, Moreira RR, Zarpellon MN, Menegucci TC, Cardoso CL (2016). IS*Aba1*/*bla*_OXA-23_: A serious obstacle to controlling the spread and treatment of *Acinetobacter baumannii* strains. Am J Infect Control.

[32] Decousser JW, Jansen C, Nordmann P, Emirian A, Bonnin RA, Anais L, et al. Outbreak of NDM-1-producing *Acinetobacter baumannii* in France, January to May 2013. Eurosurveillance. 2013;18. doi:10.2807/1560-7917.ES2013.18.31.20547 10.2807/1560-7917.es2013.18.31.20547 23929226

[33] Mathlouthi N, El Salabi AA, Ben Jomàa-Jemili M, Bakour S, Al-Bayssari C, Zorgani AA (2016). Early detection of metallo-β-lactamase NDM-1- and OXA-23 carbapenemase-producing *Acinetobacter baumannii* in Libyan hospitals. Int J Antimicrob Agents.

[34] Toleman MA, Spencer J, Jones L, Walsh TR (2012). *bla*_NDM-1_ is a chimera likely constructed in *Acinetobacter baumannii*. Antimicrob Agents Chemother.

[35] Ramoul A, Loucif L, Bakour S, Amiri S, Dekhil M, Rolain JM. Co-occurrence of *bla*_NDM-1_ with *bla*_OXA-23_ or *bla*_OXA-58_ in clinical multidrug-resistant *Acinetobacter baumannii* isolates in Algeria. J Glob Antimicrob Resist. 2016;6:136–41. 10.1016/j.jgar.2016.05.003 27530856

[36] Rafei R, Pailhoriès H, Hamze M, Eveillard M, Mallat H, Dabboussi F (2015). Molecular epidemiology of *Acinetobacter baumannii* in different hospitals in Tripoli, Lebanon using *bla*_OXA-51-like_ sequence based typing. BMC Microbiol.

[37] Bakour S, Touati A, Bachiri T, Sahli F, Tiouit D, Naim M (2014). First report of 16S rRNA methylase ArmA-producing *Acinetobacter baumannii* and rapid spread of metallo-β-lactamase NDM-1 in Algerian hospitals. 2014. J Infect Chemother.

[38] Pasanen T, Koskela S, Mero S, Tarkka E, Tissari P, Vaara M (2014). Rapid molecular characterization of *Acinetobacter baumannii* clones with rep-PCR and evaluation of carbapenemase genes by new multiplex PCR in hospital district of Helsinki and Uusimaa. Plos One.

