# Supplementary material for: Co-existence of blaOXA-23 and blaNDM-1 genes of Acinetobacter baumannii isolated from Nepal: antimicrobial resistance and clinical significance
Source: Antimicrob Resist Infect Control. 2017 Feb 7;6:21. doi: 10.1186/s13756-017-0180-5 (PMC5297125; doi:10.1186/s13756-017-0180-5)
Supplement: Additional file 1: Table S1. — Type of clinical specimen, ward, antibiotic susceptibility patterns, rep-PCR types, resistance genes and MIC of 44 A. baumannii isolates. (DOCX 23 kb) [file 13756_2017_180_MOESM1_ESM.docx]

**Supplemental data**

**Table 1S: Type of clinical specimen, ward, antibiotic susceptibility patterns, rep-PCR types, resistance genes and MIC of 44 *A. baumannii* isolates.**

| **Isolate** | **Specimen** | **Rep-PCR typing** | **Site** | **Antibiotic Resistance genes** | | | | | | | **Resistance patterns** | **MICs (µg/ml).** | | | |
| --- | --- | --- | --- | --- | --- | --- | --- | --- | --- | --- | --- | --- | --- | --- | --- |
|  |  |  |  | **OXA-23** | **OXA-24** | **OXA-51** | **OXA-58** | **NDM-1** | **ADC** | **aphA6** |  | **CAZ** | **IPM** | **TG** | **CL** |
| AB-1 | sputum | A | ICU | + | - | + | - | - | + | + | AK/CTX/CAZ/CRO/CIP/CN/  IPM/MEM/TMX/PIP | >256 | >16 | 2.5 | 2.0 |
| AB-2 | sputum | A | ICU | + | - | + | - | - | + | + | AK/CTX/CAZ/CRO/FEP/CIP/CN/IPM/MEM/TMX/TE/PIP | >256 | >32 | 2.1 | 0.91 |
| AB-3 | TA | B | ICU | + | - | + | - | - | + | - | CTX/CAZ/CRO/FEP/CIP/CN/  IPM/MEM/TMX/PIP | >256 | >32 | 1.9 | 0.27 |
| AB-4 | urine | B | GW | + | - | + | - | - | + | - | CTX/CAZ/CRO/FEP/CIP/CN/  IPM/MEM/TMX/PIP | >256 | >32 | 3.2 | 0.32 |
| AB-5 | sputum | C | POW | + | - | + | - | - | + | - | CTX/CAZ/CRO/FEP/CIP/CN/  IPM/MEM/TMX/PIP | >256 | >32 | 3.1 | 0.77 |
| AB-6 | sputum | C | ICU | + | - | + | - | - | + | - | CTX/CAZ/CRO/FEP/CIP/IPM/MEM/TMX/PIP | >256 | >24 | 1.8 | 0.41 |
| AB-7 | sputum | B | ICU | + | - | + | - | - | + | - | CTX/CAZ/CRO/FEP/CIP/CN/  IPM/MEM/TMX/PIP | >256 | >32 | 3.9 | 0.46 |
| AB-8 | pus | D | GW | + | - | + | - | - | + | + | AK/CTX/CAZ/CRO/FEP/CIP/  IPM/MEM/TMX/TE/PIP | >256 | >32 | 2.0 | 0.54 |
| AB-9 | sputum | D | GW | + | - | + | - | - | + | + | AK/CTX/CAZ/CRO/FEP/CIP/  IPM/MEM/TMX/PIP | >256 | >32 | 2.0 | 0.88 |
| AB-10 | TA | B | ICU | + | - | + | - | - | + | - | AK/CTX/CAZ/CRO/FEP/CIP/  IPM/MEM/TMX/TE/PIP | >256 | >32 | 2.1 | 0.94 |
| AB-11 | sputum | B | ICU | + | - | + | - | - | + | - | AK/CTX/CAZ/CRO/CIP/CN/  IPM/MEM/TMX/PIP | >256 | >32 | 3.5 | 0.81 |
| AB-12 | pus | D | GW | + | - | + | - | - | + | - | AK/CTX/CAZ/CRO/FEP/CIP/CN/IPM/MEM/TMX/PIP | >256 | >32 | 3.4 | 0.67 |
| AB-13 | CT | A | GW | + | - | + | - | - | + | + | AK/CTX/CAZ/CRO/FEP/CIP/CN/IPM/MEM/TMX/TE/PIP | >256 | >24 | 2.1 | 0.88 |
| AB-14 | TA | B | ICU | + | - | + | - | + | + | + | CTX/CAZ/CRO/FEP/CIP/IPM/MEM/PIP | >256 | >32 | 3.2 | 0.61 |
| AB-15 | sputum | B | POW | + | - | + | - | - | + | - | CTX/CAZ/CRO/FEP/CIP/CN/  IPM/MEM/TMX/TE/PIP | >256 | >32 | 3.0 | 0.54 |
| AB-16 | sputum | C | POW | + | - | + | - | - | + | - | CTX/CAZ/CRO/FEP/CIP/IPM/MEM/TMX/TE/PIP | >256 | >32 | 2.4 | 0.78 |
| AB-17 | sputum | C | ICU | + | - | + | - | + | + | - | CTX/CAZ/CRO/FEP/CIP/CN/  IPM/MEM/TMX/TE/PIP | >256 | >32 | 1.7 | 0.79 |
| AB-18 | sputum | B | ICU | + | - | + | - | - | + | - | AK/CTX/CAZ/CRO/FEP/CIP/CN/IPM/MEM/TMX/PIP | >256 | >32 | 2.8 | 0.13 |
| AB-19 | sputum | D | ICU | + | - | + | - | - | + | - | AK/CTX/CAZ/CRO/FEP/CIP/  IPM/MEM/TMX/TE/PIP | >256 | >32 | 2.8 | 0.32 |
| AB-20 | sputum | B | ICU | + | - | + | - | - | + | - | CTX/CAZ/CRO/FEP/CIP/CN/  IPM/MEM/TMX/PIP | >256 | >32 | 3.4 | 0.37 |
| AB-21 | sputum | E | ICU | + | - | + | - | - | + | - | AK/FEP/CIP/IPM/MEM/TMX/TE/PIP | >256 | >32 | 1.9 | 0.41 |
| AB-22 | TA | C | POW | + | - | + | - | + | + | - | CTX/CAZ/CRO/FEP/CIP/CN/  IPM/MEM/TMX/TE/PIP | >256 | >32 | 2.1 | 0.23 |
| AB-23 | pus | C | GW | + | - | + | - | - | + | - | CTX/CAZ/CRO/FEP/CIP/IPM/MEM/TMX/TE/PIP | >256 | >32 | 2.3 | 0.63 |
| AB-24 | CT | D | GW | + | - | + | - | - | + | + | AK/CTX/CAZ/CRO/FEP/CIP/  IPM/MEM/TMX/PIP | >256 | >32 | 2.0 | 0.63 |
| AB-25 | pus | E | ICU | + | - | + | - | - | + | + | No resistant | 4.0 | 1.0 | 1.8 | 1.19 |
| AB-26 | TA | D | ICU | + | - | + | - | - | - | + | CTX/CAZ/CRO/FEP/CIP/IPM/MEM/TMX/PIP | >256 | >32 | 2.8 | 0.41 |
| AB-27 | TA | C | ICU | + | - | + | - | - | + | - | CTX/CAZ/CRO/FEP/CIP/CN/  IPM/MEM/ TMX/TE/PIP | >256 | >32 | 3.1 | 0.41 |
| AB-28 | sputum | C | POW | + | - | + | - | + | + | - | CTX/CAZ/CRO/FEP/CIP/CN/  IPM/MEM/ TMX/TE/PIP | >256 | >32 | 2.3 | 0.48 |
| AB-29 | sputum | A | POW | + | - | + | - | + | + | + | AK/CTX/CAZ/CRO/FEP/CIP/  IPM/MEM/TMX/PIP | >256 | >32 | 3.2 | 0.51 |
| AB-30 | sputum | C | ICU | + | - | + | - | - | + | - | CTX/CAZ/CRO/FEP/CIP/IPM/MEM/TMX/TE/PIP | >256 | >32 | 3.0 | 0.43 |
| AB-31 | sputum | C | ICU | + | - | + | - | - | + | + | CTX/CAZ/CRO/CN/FEP/CIP/  IPM/MEM/ TMX/TE/PIP | >256 | >32 | 1.6 | 0.47 |
| AB-32 | sputum | C | ICU | + | - | + | - | - | + | + | CTX/CAZ/CRO/FEP/CIP/IPM/MEM/TMX/TE/PIP | >256 | >32 | 1.9 | 0.91 |
| AB-33 | CT | F | ICU | + | - | + | - | - | + | - | CTX/CAZ/CRO/FEP/CIP/CN/  IPM/MEM/ TMX/TE/PIP | >256 | >32 | 2.5 | 0.68 |
| AB-34 | CT | D | ICU | + | - | + | - | - | - | + | AK/CTX/CAZ/CRO/FEP/CIP/ IPM/MEM /PIP | >256 | >32 | 2.4 | 0.95 |
| AB-35 | TA | D | ICU | + | - | + | - | - | - | - | CTX/CAZ/CRO/FEP/CIP/IPM/MEM/TMX/PIP | >256 | >32 | 2.4 | 0.84 |
| AB-36 | sputum | D | GW | + | - | + | - | - | + | - | CTX/CAZ/CRO/CIP/IPM/MEM/TMX/PIP | >256 | >32 | 2.0 | 0.7 |
| AB-37 | sputum | G | GW | + | - | + | - | - | + | + | AK/CTX/CAZ/CRO/FEP/CIP/  IPM/MEM/ TMX/PIP | >256 | >32 | 2.8 | 0.68 |
| AB-38 | TA | D | POW | + | - | + | - | + | + | + | AK/CTX/CAZ/CRO/FEP/CIP/  IPM/MEM/ TMX/TE/PIP | >256 | >32 | 2.3 | 0.32 |
| AB-39 | TA | A | POW | + | - | + | - | - | + | + | AK/CTX/CAZ/CRO/FEP/CIP/CN/IPM/ MEM/TMX/TE/PIP | >256 | >32 | 3.3 | 0.59 |
| AB-40 | sputum | D | ICU | + | - | + | - | - | + | + | AK/CTX/CAZ/CRO/FEP/CIP/  IPM/MEM/ TMX/PIP | >256 | >32 | 1.9 | 0.71 |
| AB-41 | sputum | D | ICU | + | - | + | - | - | - | + | AK/CTX/CAZ/CRO/FEP/CIP/CN/IPM/ MEM/TMX/PIP | >256 | >32 | 2.6 | 0.98 |
| AB-42 | sputum | C | ICU | + | - | + | - | - | + | - | CTX/CAZ/CRO/FEP/CIP/IPM/MEM/TMX/TE/PIP | >256 | >32 | 3.1 | 0.26 |
| AB-43 | sputum | C | ICU | + | - | + | - | - | + | - | CTX/CAZ/CRO/FEP/CIP/CN/  IPM/MEM/ TMX/TE/PIP | >256 | >32 | 3.0 | 0.41 |
| AB-44 | sputum | C | ICU | + | - | + | - | - | + | - | CTX/CAZ/CRO/FEP/CIP/CN/  IPM/MEM/ TMX/TE/PIP | >256 | >32 | 2.3 | 0.49 |

TA, tracheal aspirates; CT, catheter tip; ICU, intensive care unit; GW, General ward; POW, post-operative ward; AK, amikacin, CTX, cefotaxime; IPM, imipenem; FEP, cefepime; CIP, ciprofloxacin; CRO, ceftriaxone; CN, gentamicin; MEM, meropenem; TMX, trimethoprim/sulfamethoxazole; TE, tetracycline; PIP, piperacillin/tazobactam; CAZ, ceftazidime.
